# 3D- Printed Poly(ε-caprolactone) Scaffold Integrated with Cell-laden Chitosan Hydrogels for Bone Tissue Engineering

**DOI:** 10.1038/s41598-017-13838-7

**Published:** 2017-10-17

**Authors:** Liang Dong, Shao-Jie Wang, Xin-Rong Zhao, Yu-Fang Zhu, Jia-Kuo Yu

**Affiliations:** 1 School of Materials Science and Engineering, University of Shanghai for Science and Technology, 516 Jungong Road, Shanghai, 200093 P.R. China; 2 Institute of Sports Medicine, Beijing Key Laboratory of Sports Injuries, Peking University Third Hospital, Beijing, 100191 P.R. China; 3 Department of Joint Surgery, Zhongshan Hospital of Xiamen University, Xiamen, China; 4 Medical and Health Analysis Center, Health Science Center, Peking University, Beijing, 100191 P.R. China

## Abstract

Synthetic polymeric scaffolds are commonly used in bone tissue engineering (BTE) due to their biocompatibility and adequate mechanical properties. However, their hydrophobicity and the lack of specific cell recognition sites confined their practical application. In this study, to improve the cell seeding efficiency and osteoinductivity, an injectable thermo-sensitive chitosan hydrogel (CSG) was incorporated into a 3D-printed poly(ε-caprolactone) (PCL) scaffold to form a hybrid scaffold. To demonstrate the feasibility of this hybrid system for BTE application, rabbit bone marrow mesenchymal stem cells (BMMSCs) and bone morphogenetic protein-2 (BMP-2) were encapsulated in CSG. Pure PCL scaffolds were used as controls. Cell proliferation and viability were investigated. Osteogenic gene expressions of BMMSCs in various scaffolds were determined with reverse transcription polymerase chain reaction (RT-PCR). Growth factor releasing profile and mechanical tests were performed. CCK-8 assay confirmed greater cell retention and proliferation in chitosan and hybrid groups. Confocal microscopy showed even distribution of cells in the hybrid system. After 2-week osteogenic culture *in vitro*, BMMSCs in hybrid and chitosan scaffolds showed stronger osteogenesis and bone-matrix formation. To conclude, chitosan/PCL hybrid scaffolds are a favorable platform for BTE due to its capacity to carry cells and drugs, and excellent mechanical strength.

## Introduction

Massive bone defect is a great challenge for both patients and orthopaedic surgeons. Among the available therapeutic strategies for promoting healing of large bone defect, bone tissue engineering (BTE) approach is promising by using scaffolds of synthetic or natural biomaterials that promote proliferation, and differentiation of bone cells^1^. A variety of bone grafts and bone substitutes are available to treat different types of bone defects. Although autografts are the optimal material, but related to a limited supply and donor-site morbidity^2^. Allogeneic bone grafts are concerned due to limited osteoinductivity and disease transmission^3^. There is increased interest in using tissue engineering to enhance bone repair and cell-based gene delivery is becoming a primary approach in BTE^4^. The use of stem cells, in particular, the BMMSCs have been the most popular in cartilage and bone repair^5^. Bone scaffolds are typically made of porous degradable materials that provide the mechanical support during the formation of repair tissue^6^. The key requirements of an ideal BT scaffold should include: macroporosity >100 μm, microporosity <20 μm, interconnected open pores for cell and tissue invasions, sufficient mechanical strength and appropriate *in vivo* degradation time to match the speed of tissue in-growth^7^. Actually, there are various conventional and manual techniques used for scaffold fabrication such as solvent casting and gas forming. However, these methods can only produce scaffolds with randomly-formed internal architecture which is highly dependent on the process^8^. Solid free-form fabrication (SFF)based techniques are probably the most widely used for the fabrication of 3D interconnected porous scaffolds^9^. Fused deposition modeling (FDM) is the first SFF technique based on extruding a molten polymer which requires the use of thermoplastic material. PCL is commonly used for bone scaffolds due to its high mechanical strength, the low melting temperature and glass transition temperature, as well as high thermal stability^10^. However, synthetic materials usually have limited cell affinity, due to their hydrophobicity and lack of surface cell recognition sites^11,12^. On the other hand, naturally derived polymers, such as chitosan and collagen, can potentially interact with cells via cell surface receptors and regulate cell function. Natural polymer derived hydrogels are capable of retaining large amounts of water and closely similar to living tissues. In addition, they can also provide chemical cues for cells by incorporation of bioactive molecules^13,14^. Therefore, they have been widely used as cell and drug carrier in tissue engineering^15^. Chitosan is the partial deacetylated derivative of chitin and is one of the most widely used natural polymers for the fabrication of biomaterials for BTE^16,17^. An increased basicity will lead to a gel-like precipitation of chitosan by neutralization of the amine groups. The ease of gelation without toxic additives and a sol/gel transition upon injection in the body are desirable for *in situ* injection for bone and cartilage defects^18^. However, the mechanical strength of the aforementioned natural polymers may be inferior and subject to variable enzymatic host degradation^19,20^, which hampered the application of chitosan in load-bearing bone defect.

In the current work, we integrated 3D-printed porous PCL scaffolds with chitosan thermogels to form a hybrid scaffold for BTE. The porous PCL scaffolds were fabricated by FDM using bio-printer. Thermo-sensitive chitosan hydrogel was obtained by blending chitosan glycerol phosphate disodium as previously reported^21^. Rabbit bone marrow (BM) MSCs were isolated as the osteogenic cells in the current study.

The aim of this *in vitro* study was to evaluate whether the hybrid scaffold could improve the proliferation and enhance osteogenesis of BMMSCs. We hypothesized the chitosan/PCL scaffolds can improve cell seeding efficacy, osteoinductivity, and give excellent mechanical stiffness, when compared to chitosan-thermogel or PCL scaffold alone.

## Results and Discussions

Hydrogels are highly hydrated three-dimensional networks of polymers that facilitate cells adhesion, proliferation and differentiation, and allow easy exchange of nutrients and metabolic waste with surrounding tissues. They have been used as a soft scaffold or as cell carrier for tissue engineering^18,22–24^. Among various hydrogels, natural polymer derived thermo-sensitive chitosan hydrogel has recently been paid much attention due to its advantages such as ease of use, efficiency of cell encapsulation and drug delivery^25–27^. For scaffold implantation in a load-bearing bone defect, the scaffold should possess adequate mechanical strength to withstand the mechanical requirement. The major limitation of chitosan hydrogel is the inherent mechanical weakness, which has restricted *in vivo* application for bone repair. In this study, chitosan thermogel was incorporated into porous PCL scaffolds fabricated by RP technologies. By loading CSG into the PCL scaffolds as shown in this work, advantages of CSG are preserved, while the mechanical performance are warranted by the PCL scaffolds, which entitle this hybrid scaffold competent for potential BTE scaffold application.

**Figure 1:**
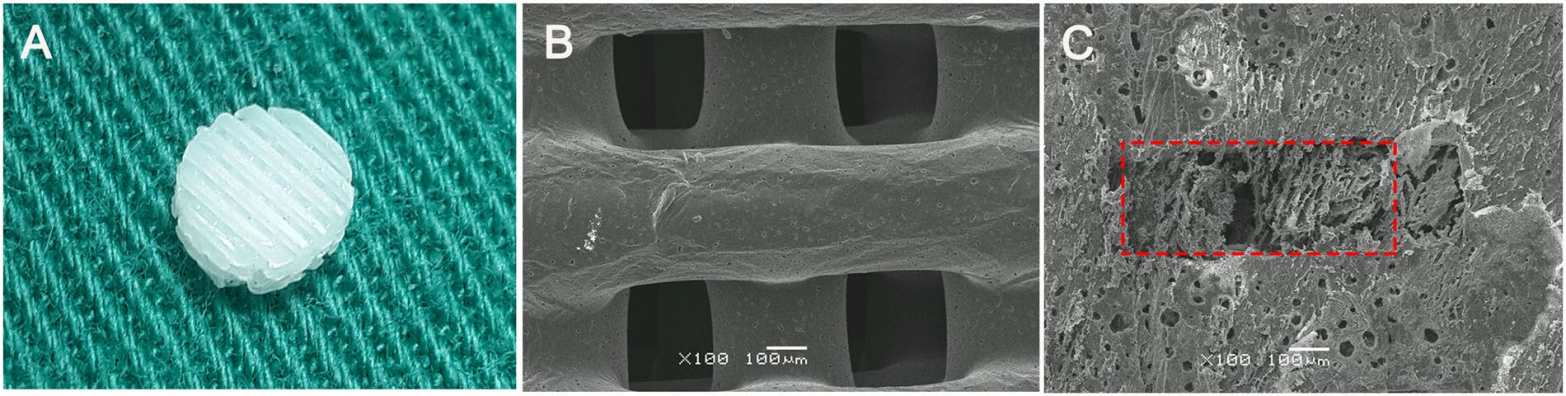
The gross appearance of 3D PCL scaffold (**A**); SEM images of PCL scaffold (**B**) and hybrid scaffold (**C**) the red rectangle showing the pores of PCL scaffold filled with the chitosan gel.

Using FDM technique we fabricated a porous PCL scaffold with open and interconnected pores, as well as excellent mechanical strength. PCL scaffolds exhibited well-controlled and interconnected porous structures. (Fig. 1A–C). The mean pore size of PCL scaffolds was 325.2 ± 26.3 μm. The mean porosity of scaffolds was 62.4 ± 0.23% The pores of hybrid scaffold were filled with chitosan hydrogel effectively.

**Figure 2:**
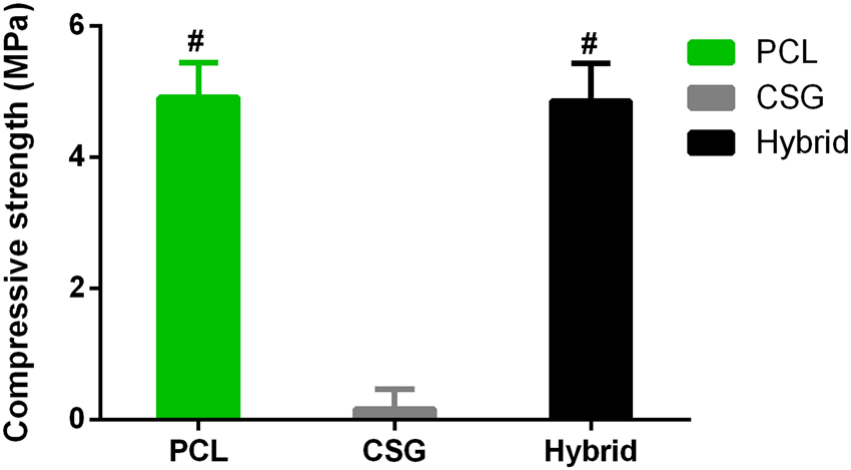
Mechanical strength of various scaffolds. Results are expressed as mean ± SD, n = 4.

The hybrid scaffold should allow for the in-growth of neo-tissue while provide a temporary physically supporting environment for BMMSCs to differentiate *in vivo*. CSG displayed the weakest unconfined compressive strength compared to other scaffolds (Fig. 2). No significant difference of unconfined compression modulus was found between PCL scaffold and hybrid scaffold (P > 0.1). The compressive modulus of the hybrid scaffold significantly increased when compared to chitosan gel alone, after the PCL scaffolds were supplemented. The compressive strength possessed by PCL and hybrid scaffold are about 6.7 MPa (similar to cancellous bone tissue^28^) and is much higher than that of other polymeric scaffolds made of PLGA or PCL^29,30^.

**Figure 3:**
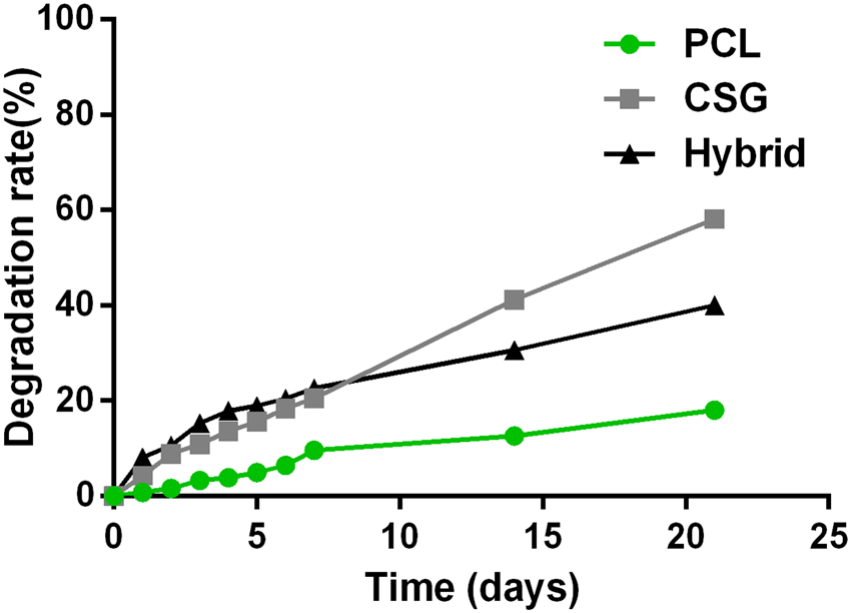
*In vitro* degradation rate of various scaffolds in PBS (n = 5).

The results of *in vitro* degradation (Fig. 3) indicated a low biodegradation ratio for the PCL scaffolds, which is a barrier for the generation of new bone. On the contrary, approximately 60% of CSG has degraded after 3 weeks of immersion in PBS. Preferably, the weight loss ratio of the hybrid scaffold increased gradually and is less than that of CSG due to the remaining PCL. The rapid degradation of CSG in hybrid scaffolds enables new tissue formation and the sustained release of encapsulated bioactive molecules while the remaining PCL is expected to provide additional mechanical support for neo-tissue.

**Figure 4:**
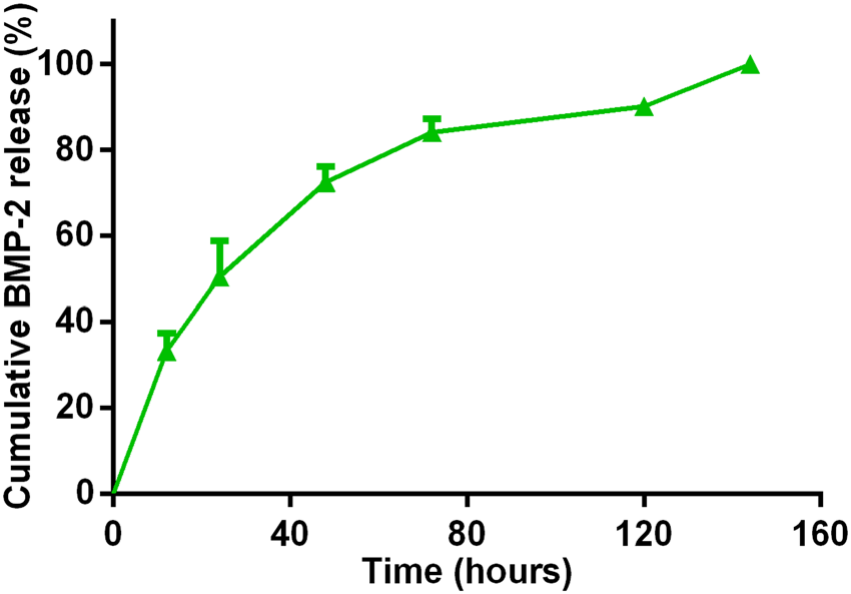
Controlled release kinetics of rhBMP-2 from chitosan gels. Results are expressed as mean ± SD, n = 3.

A sustained release of rhBMP-2 from CSG was observed over 144 h during *in vitro* incubation (Fig. 4). This confirmed the potential of CSG as an osteogenic scaffold material. Although the release of the rhBMP2 was initially 30% at 12 h, the rhBMP2 concentration continued to increase until the end point at 6 days. This is beneficial for osteogenesis compared to traditional osteogenic induction method with osteogenic growth factors degrading fast when directly mixed with the medium.

**Figure 5:**
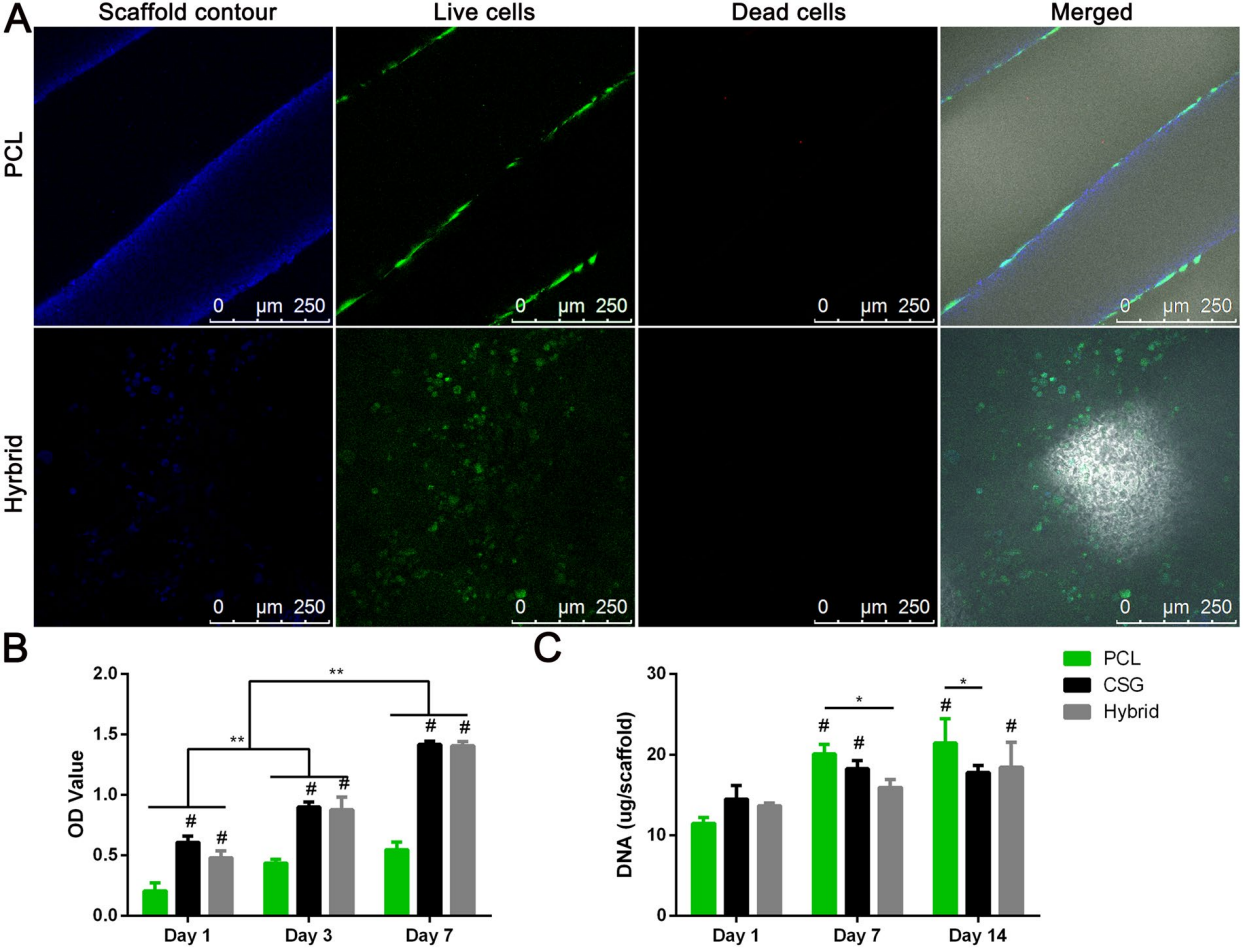
The representative images of attachment, viability and distribution of BMMSCs in composite scaffolds (**A**). Blue fluorescence represents the contours of scaffolds, merged images included bright filed views to show the pores of the scaffolds. Confocal microscopic images of Live/Dead staining demonstrated cell viability of three groups after 72 hours of culture. The distribution of MSCs on the PCL strands and on the pores and strands of hybrid scaffolds were clearly displayed (Red, dead cells; green, live cells; Scale bar = 250 μm). CCK-8 assay showed that the number of cells in the three groups increased over time (**B**). DNA content in the various scaffolds during osteogenic culture indicating slow proliferation while MSCs differentiating into osteoblasts. Results are expressed as mean ± SD, (n = 3, *^,#^P < 0.05, **^,##^P < 0.01; ^#^compared to PCL group in (**B**), and compared to day 1 in (**C**)).

As shown by Live-Dead Assay (Fig. 5A), the hybrid scaffold is favourable for cell survival and retention. After 72 hours of culture in growth medium, BMMSCs were viable in both PCL scaffolds and hybrid scaffolds with neglectable number of dead cells, yet in a different distributing pattern. BMMSCs displayed elongated morphology on PCL strands and only a limited amount of BMMSCs attached on the PCL strands which is related to hydrophobicity of PCL and the porous structure that is not supporting cell adhesion and retention. Meanwhile, cells encapsulated in hybrid scaffolds evenly distributed not only in the pores and but also spreading on the surfaces of PCL scaffolds. This finding was also consistent with the results of CCK assay which demonstrated that all cell-scaffold constructs showed an active proliferation during 7 days of *in vitro* culture (Fig. 5B). However, the number of cells in gels and gel-filled scaffolds were greater that in PCL scaffold at all time points. The number of cells were not significantly different between CSG group and hybrid group. These observations suggested excellent biocompatibility of hybrid scaffold compared to hydrophobic PCL alone, which is due to the highly hydrated environment provided by the hybrid scaffolds. Therefore, the hybrid scaffold displayed a biomimetic micro-environment for better cell retention, growth and even distribution, while having the desired mechanical strength for bone TE. DNA content measurement showed a different trend of cell proliferation when MSCs were cultured in osteogenic media (Fig. 5C). The PCL group displayed earlier and faster cell proliferation than CSG and hybrid groups. The slow cell proliferation reflected terminal osteogenesis in CSG and hybrid groups. It has been suggested that the presence of induction media held back cell proliferation as MSCs do not proliferate when undergoing differentiation^31,32^.

**Figure 6:**
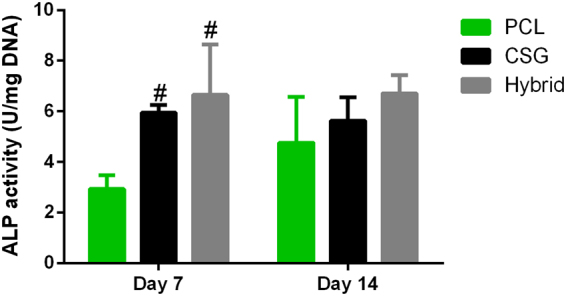
The ALP activity levels were measured and normalized to DNA content. Results are expressed as mean ± SD, n = 4.

Most importantly, BTE scaffolds should be ideally instructive for cell differentiation. To evaluate the osteogenic ability of seeded BMMSCs in various scaffolds, we compared the ALP activity of MSCs in three groups of constructs (Fig. 6). The ALP activities of MSCs in CSG and hybrid groups were significantly higher than that in PCL group at 7 days, and the ALP activity levels maintained until 14 days. However, the ALP activities among three groups were not significantly different at 14 days (p > 0.05). These results suggested that chitosan incorporated into PCL scaffolds enhanced osteogenesis of BMMSCs as early as 7 days, compared to PCL scaffolds alone.

**Figure 7:**
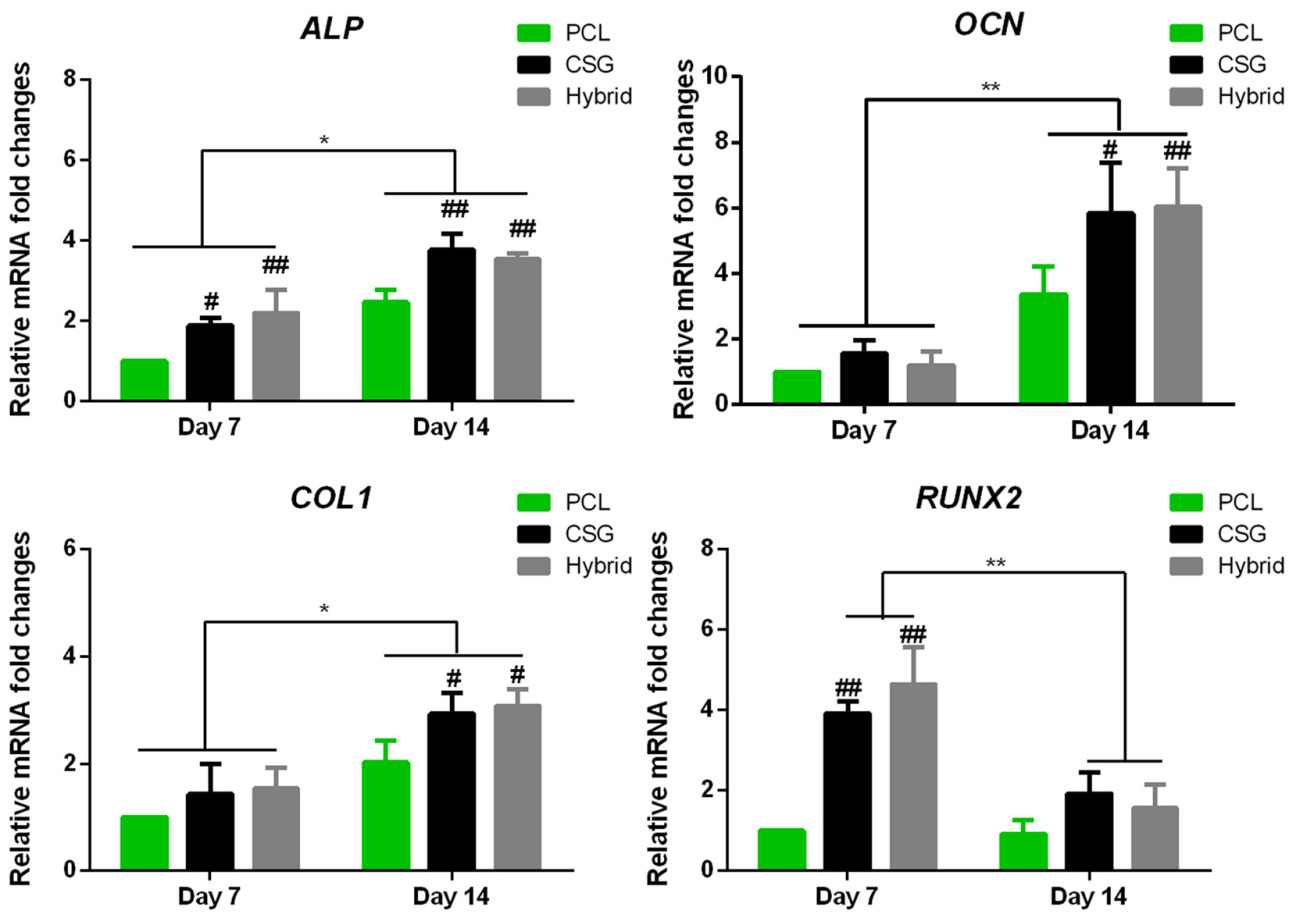
Expression of the osteogenesis-specific genes alkaline phosphatase (ALP), collagen type I (COL1), osteocalcin (OCN), and Runt-related transcription factor (RUNX2) within the scaffolds. Results are expressed as mean ± SD. (n = 3, *^,#^P < 0.05, **^,##^P < 0.01; ^#^represents significance compared to PCL group at the same time points).

Bone-specific gene expressions were also examined during *in vitro* osteogenic induction of MSC-seeded scaffolds. Osteogenic differentiation marker genes were found different between days 7 and 14 (Fig. 7). Expressions of ALP, COL1 and OCN of all groups were significantly upregulated at day 14 compared to that at day 7. Whereas RUNX2 was increased and peaked at day 7 and then decreased at day 14. RUNX2 is one of the essential transcription factors for osteoblast differentiation^33,34^. The RUNX2 expression in hybrid and CSG groups peaked at day 7 prior to other osteoblastic markers (ALP, COL1 and OCN). And the level of RUNX2 was markedly higher than that in PCL group. Accordingly, at 14 days, chitosan and chitosan-filled PCL constructs expressed remarkably greater levels of ALP, OCN, and COL1 compared to pure PCL scaffolds. This is consistent with the mechanism that RUNX2 regulated the expression of OCN, bone sialoprotein (BSP), COL1 at an early stage^35^.

In addition, gene expressions varied among BMMSCs-seeded constructs. At day 7, CSG and hybrid groups expressed higher levels of RUNX2 and ALP than PCL groups. The level of ALP expression of CSG and hybrid groups remained higher at day 14, when compared to PCL group. The expressions of OCN and COL1 were similar among all groups at day 7. However, at day 14, higher levels of OCN and COL1 were found in CSG and hybrid groups than that in PCL groups. As a result, hybrid and CSG groups showed the strongest expression of all bone-specific genes. The gene expression levels were similar between hybrid and chitosan gel groups suggesting that the chitosan is the main factor contributing to the enhanced osteogenesis and the PCL scaffold did not influence *in vitro* osteogenesis. However, the result may differ when the hybrid scaffold is applied *in vivo* where mechanical load also plays a role in the regulation of cell differentiation.

**Figure 8:**
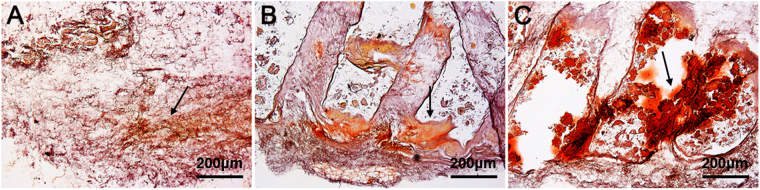
Representative images of ARS showing calcium deposition by MSCs-seeded scaffolds in nude mice, CSG (**A**), PCL (**B**), hybrid (**C**), black arrow indicating calcified bone matrix. scale bar = 200 μm.

Furthermore, *in vivo* bone matrix formation after subcutaneous implantation of various MSC-seeded scaffolds was studied in a nude mice model. Alizarin Red Staining (ARS) showed only disperse calcium deposition in CSG group, and limited calcium deposition on the scaffold fibres in PCL group. In comparison, larger area of ARS was present in the pores and fibres of hybrid scaffolds (Fig. 8). This also demonstrated that even cell distribution, better cell retaining and rigid support of porous hybrid scaffolds have improved the formation and calcification of bone matrix.

Moreover, it has been suggested that pore sizes >300 μm are beneficial for enhanced new bone formation and the formation of capillaries. Because small pores favoured hypoxic conditions and would induce chondrogenesis, whereas larger pores would lead to better angiogenesis and direct osteogenesis^36^. Therefore, we used the FDM technique to selectively designed the PCL scaffolds of 300 μm pore sizes to meet the above-mentioned requirement of bone scaffolds. Considering the slow degradation of PCL, it is reasonable to presume that, after the degradation of CSGs, the porous scaffolds with recommended 300 μm pore size and 60% porosity will remain beneficial for matter exchange and in-growth of cells and neo-tissues.

Our study is not without limitations. Firstly, we did not study the influence of pore sizes of PCL scaffolds on overall mechanical strength and cell retaining capacity. However, we based our choice of pore size on an elaborated review which recommended scaffolds with mean pore sizes (300 μm) for osteogeneiss and vascularization^37^. In addition, the current study is a preliminary study focusing on the effect of chitosan thermogel on *in vitro* osteogenic differentiation of MSCs. Further study with *in vivo* application of the hybrid scaffolds in load-bearing bone defects should be performed.

## Methods

### 3D-Bioprinting of PCL Scaffolds

Medical grade PCL (number-average molecular weight (Mn) = 84,200 Da and melting point (MP) (53.5 °C) was provided by Daigang Biomaterials (Shandong, PR China). PCL was melted in the printing cartridge at a temperature of 130 °C. 3 D scaffolds were fabricated using a 3-D Bioplotter™ (EnvisionTEC GmbH, Germany), pressurized at 1.5–3.0 bar and extruded to produce a melt filament through a heated metal micro-nozzle (22 G, 400 μm). The movement of the micro-nozzle along the X, Y, and Z axis were controlled using a CAD (Delta Tau Data Systems Inc., Chatsworth, CA, USA) file. The nozzle speed was set at 1–3 mm·s^−1^. The strands were dispensed layer by layer, forming a 0°- to 90°- oriented junctions with fiber spacing of 300 μm and a Z axis interlayer increment of 300 μm. The printed scaffolds measuring 10 × 10 × 2 mm. was punched into cylindrical discs with a 6-mm diameter corneal trephine. The resultant scaffold was shown in Fig. 1 All scaffolds types were immersed in ethanol for 48 hours for disinfection and then rinse twice with PBS before further examination.

### Chitosan Thermogel Preparation

Chitosan (degree of deacetylation >90%, viscosity 130 mPa for 1% (w/v) solution; Shandong Jinhu Company, China) was dissolved in 0.1 M acetic acid to obtain a 3 wt% chitosan solution. An 8 wt% β-glycerophosphate solution (Baierdi Company, Beijing, China) and the chitosan solution were kept at 4 °C before being mixed to obtain chitosan-GP solution. The final concentration of 2.7 wt% for chitosan and 8 wt% for GP, respectively. This mixture was used in all following experiments. Gelation of the final solution was initiated by incubation at 37 °C. The sol—gel transition behaviour of chitosan themogel was confirmed by incubation at 37 °C for 15 min to initiate gelation.

### Chitosan/PCL Hybrid Scaffolds Fabrication

Chitosan/PCL hybrid scaffolds were fabricated by impregnating the PCL scaffold in the precooled chitosan/GP solution at 4 °C. The chitosan/GP solution was dropped onto the surface of PCL scaffold in a vertically placed 0.5 mL EP tube. The EP tube was then sent for centrifugation at 4 °C to achieve a homogenous gel distribution in the porous scaffold. The scaffold was then removed from the tube and incubated at 37 °C for gelation.

### Morphology, Porosity of Scaffolds

The morphology of three groups of scaffolds were characterized with a field emission scanning electron microscope (SEM) (FESEM S-4800, HITACHI, Japan). The size of ten pores for each scaffold (n = 3) was determined using Image-Pro Plus software 6.0 (Media Cybernetics). Scaffold porosity was measured with a liquid displacement technique. W_dry_ is the dry weight of scaffolds, W_sus_ is the weight of scaffolds suspended in water, and Wsat is the weight of scaffolds saturated with water. Porosity was calculated by eq. (1).

1 $${\rm{Porosity}}\,( \% )=({{\rm{W}}}_{{\rm{sat}}}-{{\rm{W}}}_{{\rm{dry}}})/({{\rm{W}}}_{{\rm{sat}}}\,-\,{{\rm{W}}}_{{\rm{sus}}})\times 100 \% $$

### Mechanical Property of Scaffolds

Unconfined compression tests were performed on CSG, PCL scaffold, and hybrid scaffolds, using previous protocol^38^. The height and diameter of each sample was recorded for later calculations. The sample was subjected to a stress relaxation test to obtain either the equilibrium unconfined compressive strength. Four specimens of each scaffold type were loaded with a force of 0.02 N until the load cell platen came into contact with the sample. After equilibrium was achieved, stress relaxation tests were conducted with a compressive deformation of 0.06 mm/min to 10%. Samples were then released to reach equilibrium of displacement (1200s).

### Degradation of Scaffolds

Scaffolds were immersed and incubated in PBS for 0, 1, 2, 3, 4, 5, 6, 7, 14 and 21 days at 37 °C. After equilibrium in PBS, all scaffolds were weighed and recorded as W_0_. At various intervals, scaffolds were recovered and weighed (W_t_). of scaffolds was calculated as eq. (2):

2 $${\rm{Degradation}}\,{\rm{ratio}}=({{\rm{W}}}_{0}-{{\rm{W}}}_{{\rm{t}}}){/{\rm{W}}}_{{\rm{0}}}\times 100 \% $$

### *In vitro* Release Kinetics of rhBMP-2

To study its release kinetics, 50 ng of rhBMP-2 was loaded into 1 mL hydrogels prior to crosslinking. The hydrogels were then immersed incubated in media containing 1 mL phosphate buffered saline (PBS) at 37 °C. Samples of media (1 mL) were collected after 12, 24, 48, 72, 120, and 144 h, and were substituted by equal amounts of fresh PBS. Concentrations of released rhBMP-2 in the collected media samples were measured with a rhBMP-2 ELISA Kit as the manufacturer’s instructions (Cloud-Clone CORP.). The cumulative release of rhBMP-2 was then expressed as a percentage of the total release amount.

### Isolation and Culture of BMMSCs

All animal experimental protocols were approved by the Animal Care and Use Committee of Peking University and the methods in the current work were carried out in accordance with the Guide for the Care and Use of Laboratory Animals. Two-month-old adult New Zealand white rabbits were used for the isolation of BMMSCs. The isolation, culture and identification of BMMSCs were performed according to the our previous protocols^39^. BMMSCs at passage 5 were used for subsequent experiments.

### Cell Seeding and Cell-scaffold Construct Culture

BMMSCs pellet at a cell density of 5.0 × 10^5^ was mixed and re-suspended with 40 μL of chitosan/GP solution (containing 50ng/ml rhBMP-2 for gel and hybrid constructs and no rhBMP-2 for PCL scaffold) at 4 °C. Then the mixed cell-chitosan suspension was incorporated into the PCL porous scaffold and incubated at 37 °C for 15 min to form a hybrid/BMMSCs construct. The chitosan/BMMSCs construct was prepared by directly dripping BMMSCs-chitosan suspension into a 24-well plate. The PCL/BMMSCs construct was prepared by adding 40 μL of BMMSCs suspension of the same cell density to PCL scaffolds using afore-mentioned centrifugation method. The hybrid/BMMSCs, PCL/BMMSCs, and chitosan/BMMSCs constructs were then incubated in a 5% CO_2_, 37 °C incubator for 1 hour for initial cell attachment. Then the seeded scaffolds were supplied with 2 ml of fresh α-MEM supplemented with 10% FBS (Gemini BioProducts, Woodland, CA), and 1% penicillin and streptomycin (Invitrogen) and cultured at 37 °C, 5% CO_2_, and 100% humidity. For cell proliferation assay and DNA content analysis, the cell-seeded scaffolds were cultured for one week in growth medium, i.e., α-MEM. For osteogenesis induction, the cell-seeded gel or hybrid constructs were cultured in osteogenic differentiation medium (RBXMX-90021; Cyagen Biosciences Inc., Guangzhou, China) without additional rhBMP-2. To make the culture comparable, the osteogenic culture for PCL/BMMSCs constructs was the same medium supplemented with rhBMP-2 (50ng/mL). The culture medium was changed every 3 days.

### Cell Viability and Proliferation on Scaffolds

Cell viability seeded in scaffolds was evaluated using a LIVE/DEAD Viability/Cytotoxicity Kit assay (Invitrogen, Carlsbad, CA, USA) under confocal microscopy (Leica, Nussloch, Germany). The cell-seeded PCL or hybrid scaffolds were cultured in growth medium for 72 hours. Then cell-scaffold constructs (n = 3) were washed in PBS (pH 7.4) for three times. Each construct was immersed in 500 μL of PBS with 2 mM calcein AM and 4 mM ethidium homodimer-1 reagents and incubated for 2 h at 37 °C. Excitation wavelength of 568 or 488 nm was used to detect the fluorescence of ethidium homodimer-1 (dead cells = red) or calcein AM (live cells = green). Non-seeded scaffolds were also stained as blank control to avoid background effect. The proliferation activity of cells was quantified at on day 1, 3, and 7 using a Cell Counting Kit-8 assay (CCK-8; Dojindo Laboratories, Kumamoto, Japan), according to the manufacturers’ instructions. Briefly, cell-seeded scaffolds (n = 3) were gently rinsed in PBS and then submersed in a mixed solution of 10 μL of CCK-8 reagent with 90 μL of fresh medium at 37 °C for 2 h. The absorbance readings at 450 nm were observed using a plate reader.

### Biochemical Analysis

To detect the cell proliferation during *in vitro* osteogenic induction, cell seeded constructs were cultured in osteogenic differentiation medium (RBXMX-90021; Cyagen Biosciences Inc., Guangzhou, China) for 14 days. At 1, 7, and 14 days, The cell-scaffold constructs were collected and washed gentle in cold PBS (4 °C) before lysis in RIPA buffer (Beyotime, Jiangsu, China). The aliquots of the sample digestion were used for DNA content measurements. DNA content was measured using Hoechst 33258 working solution (2 μg/mL) for fluorometric anaylysis. The fluorescence was read at 360 nm for excitation and 460 nm for emission. The DNA content was normalized with a standard curve of calf thymus DNA (Sigma, St Louis, Missouri, USA).

Part of the lysis solution was centrifuged, and the supernatant was used for ALP activity with p-nitrophenyl phosphate assay. The ALP activity were detected following manufacturer’s instructions (BioVision, CA, USA). The ALP activity of the test samples was calculated as umol of converted p-nitrophenol/min, and then normalized to the detected DNA content. The final ALP activity was expressed as U/mg DNA.

### Bone-specific Gene Expression Analysis

To compare the osteogenic capacity of seeded MSCs in various scaffolds, we measured the expressions of osteogenic genes alkaline phosphatase (ALP), collagen type I (COL1), osteocalcin (OCN), and Runt-related transcription factor (Runx2). Gene expressions from the MSCs cultured in the scaffolds were detected by quantitative real-time polymerase chain reaction (Q-RTPCR) as previously reported. At predesignated time points, samples (n = 3) were homogenized in Trizol Reagent (Invitrogen, Carlsbad, CA, USA) with a tissue grinder and RNA was extracted according to the manufacturer’s instructions. Isolated RNA concentration was determined by an ND-2000 spectrophotometer (Nanodrop Technologies). One microgram of RNA from each sample was reverse transcribed into cDNA using the mmLV Reverse kit (Promega, Madison, WI, USA), and RT-PCR analysis was performed using ABI 7300 real-time PCR system (Applied Biosystems, Foster City, CA, USA) with SYBR Green PCR Master Mix (Toyobo, Osaka, Japan). The relative gene expression was expressed by fold difference which was calculated as 2^ΔΔCT^. The relative expression changes in these target genes were quantified by normalizing their expression to that of housekeeping gene glyceraldehyde-3-phosphate dehydrogenase (GAPDH). The PCR primers are listed in Table 1.

**Table 1:** Primer sequences used for real-time PCR.

	Forward primers (5′-3′)	Reverse primers (5′-3′)
*Runx2*	TGATGACACTGCCACCTCTGA	GCACCTGCCTGGCTCTTCT
*OCN*	CTCCAGGGGATCCGGGTA	AAGCCCAGCGGTGCAGAGT
*COL1*	TGGCAAGAACGGAGATGACG	GCACCATCCAAACCACTGAA
*ALP*	CGACACGGACAAGAAACCCT	TGTTGTGAGCGTAGTCCACC
*GAPDH*	CCATCACCATCTTCCAGGAG	GATGATGACCCTTTTGGCTC

*Runx2*: runt-related transcription factor; OCN: osteocalcin; COL1: collagen type I; *ALP*: alkaline phosphatase; *GAPDH*: glyceraldehyde-3-phosphate dehydrogenase.

### *In vivo* Bone Matrix Deposition

To compare the osteogenic capacity of various scaffolds, calcium deposition *in vivo* was examined in nude mice after subcutaneous implantation of MSCs-seeded scaffolds. 7-week old male nude mice (Weitonglihua Animal Center, Beijing, China) were used in the study. MSCs-seeded scaffolds were cultured for 2 weeks under osteogenic conditions before implantation. Three weeks after implantation, the mice were killed and implants were sectioned and underwent ARS for calcification analysis.

### Statistical Analysis

All data were expressed as means ± standard deviation and represented at least three independent experiments. All data were analyzed using a two-way ANOVA test. P-values < 0.05 were considered significant. When ANOVA results were significant, post-hoc analysis was performed via Tukey’s multiple comparison test. All analyses were carried out using GraphPad Prism version 6.0 for Windows (GraphPad Software, San Diego, CA).

## Conclusions

Taken together, we successfully fabricated hybrid scaffold which possessed reinforced compressive strength and favorable micro-environment for cells growth and osteogenesis. We concluded that the CSG incorporated  PCLscaffold  is a promising 3D scaffold for potential *in vivo* bone and subchondral bone defect repair.
